
JOURNAL INFORMATION
==============================
NLM Title Abbreviation: JBRA Assist Reprod
Journal ID: JBRA Assist Reprod
NLM Journal ID: 101684552
Journal ID: jbra

JBRA Assisted Reproduction

ISSN: 1517-5693
EISSN: 1518-0557
Publisher: Brazilian Society of Assisted Reproduction

ARTICLE INFORMATION
==============================
PMCID: PMC5264383
PMID: 27584611
DOI: 10.5935/1518-0557.20160035
Article version: 1
Subjects: Oral Presentations

Oral Presentations: Abstracts of the 20th Annual Congress of the SBRA, Belo Horizonte/MG 14-17 September 2016

Publication date: 2016 Jul-Sep
Print publication date: 2016 Jul-Sep
Volume: 20
Issue: 3
First page: 165
Last page: 167
License: This is an Open Access article distributed under the terms of the Creative Commons Attribution License, which permits unrestricted use, distribution, and reproduction in any medium, provided the original work is properly cited.
License URL: https://creativecommons.org/licenses/by/4.0/

==============================


O-01. miR-142-3p as a biomarker of blastocyst implantation failure - a pilot study

Borges Jr. Edson 1 2
Setti, Amanda Souza 1 2
Braga Daniela P.A. F. 1 2 3
Geraldo Murilo V 4
Figueira Rita de Cássia S 1
Jr Assumpto Iaconelli 1
1 Fertility Medical Group - São Paulo/SP - Brazil
2 Instituto Sapientiae - Centro de Estudos e Pesquisa em Reprodução Assistida - São Paulo/SP - Brazil
3 Disciplina de Urologia, Área de Reprodução Humana, Departamento de Cirurgia, Universidade Federal de São Paulo/SP - Brazil
4 Departamento de Biologia Estrutural e Funcional, Instituto de Biologia, Universidade Estadual de Campinas - UNICAMP - SP. Brazil
First page: 165
Last page: 165

O-02. Non-invasive prediction of blastocyst implantation, ongoing pregnancy and live birth, by mass spectrometry lipid fingerprinting

Borges Jr. Edson 1 2
Braga Daniela P.A.F. 1 2 3
Setti Amanda Souza 1 2
Montanni Daniela A. 3
Cabral Elaine Cristina 4
Eberlin Marcos N. 4
Turco Edson G. Lo 3
Iaconelli Jr Assumpto 1
1 Fertility Medical Group - São Paulo/SP - Brazil
2 Instituto Sapientiae - Centro de Estudos e Pesquisa em Reprodução Assistida - São Paulo/SP - Brazil
3 Disciplina de Urologia, Área de Reprodução Humana, Departamento de Cirurgia, Universidade Federal de São Paulo/SP - Brazil
4 Laboratório ThoMSon de Espectrometria de Massas - Instituto de Química - UNICAMP
First page: 165
Last page: 165

O-03. Age male as interference factor in embryo quality. A retrospective study using the technique of "time lapse"

Rosário Guilherme R. F. 1
Vidal Diana S. 1
Silva Adriana V. 1
Franco Antônio C. C. 1
1 Embryolife Instituto de Medicina Reprodutiva, São José dos Campos/SP.
First page: 165
Last page: 166

O-04. Prediction of Metaphase II oocytes according to different serum Anti-Müllerian hormone (AMH) levels in antagonist ICSI cycles

Silva Joyce B da 1
Panaino Tatiana R 1
Tamm Maria A 1
Lira Paloma 1
Arêas Patricia C F 1
Mancebo Ana C A 1
Souza Marcelo M de 1
Antunes Roberto A 1
Souza Maria do Carmo B de 1
1 Fertipraxis Centro de Reprodução, Rio de Janeiro, RJ, Brazil
First page: 166
Last page: 166

O-05. Evaluation of effects of maintenance and transport in dry ice on the parameters of samples enabling cryopreserved

Til David 1
Amaral Vera L L 1
Salvador Rafael A 1
Senn Alfred 2
Paula Thais S de 3
1 Universidade do Vale do Itajaí - UNIVALI - Itajaí/SC, Brazil
2 Fondation pour l'Andrologie, la Biologie et l'Endocrinologie de la Reproduction - FABER - Lausanne, Switzerland
3 Instituto Sapientiae - São Paulo/SP - Brazil
First page: 166
Last page: 166

O-06. Live births after polar body biopsy and frozen-thawed cleavage stage embryo transfer: case report

Guimarães Fernando 1
Roque Matheus 1 2
Valle Marcello 1
Kostolias Alessandra 1
Azevedo Rodrigo A de 1
Martinhago Ciro D 3
Sampaio Marcos 4
Geber Selmo 2 4
1 ORIGEN - Center for Reproductive Medicine, Rio de Janeiro, Brazil
2 UFMG - Universidade Federal de Minas Gerais, Belo Horizonte, Brazil
3 Chromosome Genomic Medicine, São Paulo, Brazil
4 ORIGEN - Center for Reproductive Medicine, Belo Horizonte, Brazil
First page: 166
Last page: 167

O-07. Zika virus and Assisted Reproductive Techniques: to test or not to test, that is the question. Unnecessary costs? Two months post mandatory evaluation in an outbreak area, Rio de Janeiro, Brazil.

Souza Maria do Carmo B. de 1
Raupp Veronica 2
Sobrinho Fernanda 2
Menezes Mariana 2
Panaino Tatiana R 1
Tamm Maria A 1
Mancebo Ana C A 1
Costa Ana L R 1
Antunes Roberto A 1
1 Fertipraxis Centro de Reprodução Humana, Rio de Janeiro/RJ, Brazil
2 Hospital Federal da Lagoa. Rio de Janeiro/RJ, Brazil
First page: 167
Last page: 167

